# Beyond False Dichotomies in Ultra‐Processed Food Policy

**DOI:** 10.1111/1468-0009.70119

**Published:** 2026-08-14

**Authors:** ASHLEY N. GEARHARDT, KELLY D. BROWNELL, ALLAN M. BRANDT

**Affiliations:** ^1^ University of Michigan; ^2^ *Sanford School of Public Policy* Duke University; ^3^ Harvard Medical School; ^4^ Harvard Faculty of Arts & Sciences

**Keywords:** ultra‐processed foods, cigarettes, tobacco regulation, addiction, public policy, commercial determinants of health

We appreciate the thoughtful response to our article and strongly agree that improving diet quality should remain a central public health goal. Diets rich in minimally processed, fiber‐containing, and diverse foods are associated with better health outcomes and may support healthier gut microbial ecosystems.^1^ Public health should continue promoting nourishing dietary patterns and improving access to real food.

However, we are concerned that the response frames these goals as somehow separate from, or even incompatible with, addressing the addictive engineering of ultra‐processed foods (UPFs). In our view, this creates a false dichotomy.

For decades, public health messaging has encouraged people to “eat healthier.” Yet during this same period, the food environment has become increasingly saturated with industrially formulated products engineered to maximize reward, convenience, and excessive consumption. These products are designed to be rapidly consumed, intensely palatable, heavily marketed, and difficult to resist under conditions of stress, time scarcity, and repeated cue exposure. Increasingly, they are also wrapped in health‐oriented marketing related to “plant‐based,” “gut health,” “high protein,” or “functional” nutrition, further blurring distinctions between genuinely nourishing foods and products optimized primarily for overconsumption.

This is precisely why discussions of addictive design and corporate engineering remain important. Public health would never have addressed smoking‐related disease solely by encouraging people to breathe clean air while ignoring that the air itself was saturated with cigarette smoke from industrially engineered products designed and marketed to sustain compulsive use. Similarly, people attempting to follow healthier dietary guidance today must navigate food environments saturated with intentionally tempting UPFs in order to access and sustain minimally processed dietary patterns that would better support health. Encouraging healthier eating while declining to address the engineered nature and dominance of the modern UPF environment risks placing the burden entirely on individual decision‐making while leaving the underlying commercial drivers untouched.

Importantly, recognizing addictive properties in some UPFs does not negate the importance of promoting healthy dietary patterns. These approaches are complementary, not competing. Public health efforts should simultaneously increase access to affordable, nourishing foods and reduce the dominance of products designed to maximize craving, compulsive intake, and market capture. Framing the issue as either “promoting healthy diets” or “addressing addictive UPFs” obscures how industrial food environments can actively undermine people's ability to sustain nourishing eating patterns over time.

We also wish to clarify a broader misunderstanding regarding addiction science reflected in the response. The authors appear to suggest that the absence of robust evidence for withdrawal or tolerance undermines the relevance of addiction frameworks to UPFs. However, withdrawal and tolerance have not historically been viewed as necessary or sufficient evidence of addictive potential. Notably, the 1988 Surgeon General's report identifying cigarettes as addictive did not require the presence of marked withdrawal or tolerance syndromes to reach that conclusion.^2^ Indeed, because cocaine lacked classical physical withdrawal and nicotine produced tolerance that plateaued rather than escalated, both drugs were classified as merely “habituating” rather than addictive for decades.^3^, ^4^, ^5^ This debate was resolved only when the field broadened its understanding of addiction beyond an opioid‐derived tolerance‐and‐withdrawal template and toward a framework emphasizing compulsive use, craving, impaired control, continued use despite harm, and reinforcement potency.

Importantly, there is evidence of withdrawal‐ and tolerance‐related processes associated with UPFs in both animal and human studies.^6^ Individuals frequently report experiences with UPFs that mirror hallmark features of addictive disorders, including intense cravings, repeated unsuccessful attempts to cut down, loss of control, and continued consumption despite adverse consequences.^6^

Notably, the study cited in the response was not designed to assess withdrawal or tolerance in individuals exhibiting addictive eating phenotypes and therefore cannot meaningfully resolve this question.^7^ Addiction vulnerability varies substantially across individuals due to biological, developmental, psychological, and environmental factors. The absence of withdrawal‐like responses in a broadly selected sample cannot reasonably be interpreted as evidence against the addictive potential of a product category, particularly when the relevant population was not specifically studied.

More broadly, we believe public health frameworks must adequately account for the interaction between vulnerable human reward systems and highly engineered commercial products. Modern food environments are increasingly shaped by products designed to maximize consumption rather than nourishment. This does not imply that all UPFs are identical, nor that individuals lack agency. Rather, it reflects the reality that environments saturated with highly engineered products can make sustaining nourishing dietary patterns substantially more difficult at a population level.^8^

The history of tobacco control is instructive not because cigarettes and UPFs are identical products, but because both industries demonstrate how corporations can optimize products for repeated use while simultaneously emphasizing personal responsibility and scientific uncertainty. Public health progress occurred not simply because individuals were told to make healthier choices, but because environmental, regulatory, and commercial factors were also addressed.^9^

The same principle applies here. Educational efforts alone are unlikely to succeed if individuals must continually navigate a food environment dominated by products specifically engineered to stimulate craving and compulsive intake. Meaningful progress will require both promoting genuinely nourishing foods and critically examining the commercial systems and product designs that undermine them.

## Funding/Support

This project was supported by funding from Vital Strategies. Vital Strategies’ Food Policy Program is supported by a grant from Bloomberg Philanthropies. The funder had no role in the design, analysis, interpretation, or writing of this manuscript.

## Conflicts of Interest Statement

Dr. Gearhardt has provided paid and unpaid consultation to public health non‐profits and law firms on matters related to ultra‐processed food and public health. These activities did not influence the content, analysis, or conclusions of this manuscript.

The other authors declare no conflicts of interest relevant to the content of this article.

## References

[1] Placeholder: Food Industry Engineering Is Less Important Than Access to Gut Microbiota‐Accessible Foods for Preventing Chronic Disease. 10.1111/1468-0009.70111 PMC13560590 42470169

[2] Gearhardt AN , DiFeliceantonio AG . Highly processed foods can be considered addictive substances based on established scientific criteria. Addiction. 2023;118(4):589‐598. 10.1111/add.16065 36349900

[3] United States Department of Health and Human Services . The Health Consequences of Smoking: Nicotine Addiction: A Report of the Surgeon General. Rockville, MD: Office on Smoking and Health. DHHS Publication (PHS) 88‐8406; 1988.

[4] Gawin FH , Kleber HD . Evolving conceptualizations of cocaine dependence. Yale J Biol Med. 1988;61(2):123‐136. 3043925 PMC2590292

[5] Mars SG , Ling PM . Meanings & motives. Experts debating tobacco addiction. Am J Public Health. 2008;98(10):1793‐1802. 10.2105/AJPH.2007.114124 18703459 PMC2636463

[6] Gearhardt AN , Schulte EM . Is food addictive? A review of the science. Annu Rev Nutr. 2021;41(1):387‐410. 10.1146/annurev-nutr-110420-111710 34152831

[7] Hall KD , Ayuketah A , Brychta R , et al. Ultra‐processed diets cause excess calorie intake and weight gain: an inpatient randomized controlled trial of ad libitum food intake. Cell Metab. 2019;30(1):67‐77.e3. 10.1016/j.cmet.2019.05.008 31105044 PMC7946062

[8] Monteiro CA , Louzada ML , Steele‐Martinez E , et al. Ultra‐processed foods and human health: the main thesis and the evidence. Lancet. 2025;406(10520):2667‐2684. 10.1016/S0140-6736(25)01565-X 41270766

[9] Brandt AM . The Cigarette Century: the Rise, Fall, and Deadly Persistence of the Product That Defined America. Basic Books; 2007.

